# Anaplastic thyroid cancer: review of precision therapy options and advances

**DOI:** 10.1038/s41698-026-01524-9

**Published:** 2026-07-08

**Authors:** Noor Khalid, Razelle Kurzrock, Shumei Kato

**Affiliations:** 1 Southern Illinois University School of Medicine, Springfield, IL United States of America; 2 Medical College of Wisconsin, Froedtert and Medical College of Wisconsin Cancer Center and Linda T. and John A. Mellowes Center for Genomic Sciences and Precision Medicine, Milwaukee, WI United States of America; 3 Center for Personalized Cancer Therapy, University of California San Diego Moores Cancer Center, La Jolla, CA United States of America

**Keywords:** Biomarkers, Cancer, Oncology

## Abstract

Anaplastic thyroid cancer (ATC) is a rare and aggressive tumor with a historical median survival of less than six months. Most patients have advanced disease at the time of diagnosis. Traditional cytotoxic chemotherapy has not shown survival benefits. Molecular testing and next-generation sequencing are used to identify specific targets for patients with ATC. If positive for *BRAF* V600E mutation, patients can be treated with BRAF/MEK inhibitors. Other therapies include tyrosine kinase inhibitors (e.g., lenvatinib) and recently immune checkpoint inhibitors which have shown remarkable results in a subset of patients, including when used along with targeted therapy. An illustrative case of a patient with advanced *BRAF*-mutant ATC (respiratory failure on a ventilator) who recovered with a combination of the anti-PD1 nivolumab and the *BRAF V600E* targeted drug vemurafenib is shown. Further studies using biomarker-based gene- and immune-targeted agents, including in combination, are needed in ATC.

## Introduction

Anaplastic thyroid cancer (ATC) is a rare and aggressive malignancy, and most patients do not survive beyond six months of diagnosis^1^. Although ATC constitutes only 1–4% of thyroid cancers, mortality is high and accounts for 14–39% of deaths from the entire thyroid cancer histology^2^. For locoregional disease, guidelines recommend surgical resection if feasible, followed by adjuvant external beam radiation and chemotherapy^3,4^. However, a significant number of patients with ATC present with distant metastatic disease at the time of diagnosis, thus requiring systemic therapy. Traditional cytotoxic chemotherapy regimens include anthracyclines or taxanes with or without platinum-based agents. These have low response rates of about 15–20% and chemotherapy alone has not shown improvement in survival^5,6^. A multimodal strategy involving excisional surgery, EBRT, chemotherapy, and/or targeted therapy along with the addition of immunotherapy has shown improvement in overall survival over the years. This was reflected in a study from a tertiary care center that reported improvements in overall survival over decades, with 1- and 2-year survival rising from 35% and 18% in the 2003–2013 group to 59% and 42% in more recent years of 2017–2019 group^7^.

Common genetic mutations occurring in thyroid cancers include *BRAF*, *TERT* promoter, *RAS*, and *RET*^8^. Of these, *BRAF* V600E had a prevalence of 20-50% in patients with ATC studied in a systemic review^9^. *BRAF* activates downstream signaling of *MEK* and *ERK*. In 2018, the Food and Drug Administration (FDA) approved dabrafenib (BRAF inhibitor) in combination with trametinib (MEK inhibitor) for treatment of locally advanced *BRAF* V600E mutated ATC. This was based on results of phase II ROAR basket study that showed an objective response rate 56% on final analysis^10^. In the subset of ATC who do not harbor *BRAF* mutations, other agents are being studied. Single-targeted inhibitors have limited efficacy in ATC and thus multi-kinase inhibitors such as lenvatinib, sorafenib, sunitinib, and pazopanib have used in trials. In Japan, lenvatinib has been approved for treatment of ATC; with objective response rate of about 44%^11^. Immunotherapy with check-point inhibitors has also shown promising results, especially when combined with matched targeted therapy^12^. We review the literature on advances in treatment of ATC and present a patient with ATC who was end stage (intubated on ventilator) and was treated with the BRAF inhibitor vemurafenib along with PD-1-inhbitor pembrolizumab, achieving an excellent response.

## Classification of thyroid cancer (WHO 2022)

According to the 5th edition of WHO classification, thyroid neoplasms are divided into benign lesions, low-risk neoplasms, and malignant thyroid neoplasms^13^. Malignancies are stratified based on histological and molecular profiles and to reflect overall prognosis: follicular thyroid carcinoma (FTC), papillary thyroid carcinoma (PTC), invasive encapsulated follicular variant papillary thyroid carcinoma (IEFV-PTC), poorly differentiated thyroid carcinoma (PDTC), differentiated high-grade thyroid carcinoma (DHGTC), oncocytic carcinoma of thyroid (OCA), and anaplastic thyroid carcinoma (ATC)^14^. Certain sub-types are associated with specific mutations, like *BRAF* mutations are commonly found in PTC while *RAS* in IEFV-PTC and FTC. *RET* point mutations are the dominant genetic alterations in MTC while *RET* fusions occur in PTC and less commonly in poorly differentiated and ATC^15,16^. ATC is the most undifferentiated form and squamous cell carcinoma is considered a subtype of ATC^17^.

### Molecular biology of ATC

Multiple genetic alterations result in anaplastic transformation of thyroid. The most common mutations in ATC include mutation of *RAS* (activates both the RAS/RAF/MEK/ERK (MAPK) and PI3K/AKT pathways), *BRAF, TP53*, *PIK3CA*, *APC, TERT*, *RET*, and *PTEN*^18,19^. Others include mutations in cell cycle genes, SWI/SNF chromatin remodeling complex, histone modification, RAC1 as well as in mismatch repair pathway. Also, overactivity of AID/APOBEC family of cytidine deaminases and amplification of PD-L1 and PD-L2 genes may play a role in the pathogenesis of ATC^20^.

## Novel treatments for ATC

### BRAF/MEK inhibitors (Table 1)

It has been noted that the prevalence of *BRAF* mutation in anaplastic thyroid cancer varies geographically, with European studies reporting lower rates (30%) as compared to American (40–50%) and Asian (60%) studies^21–25^. The geographic variability in reported *BRAF* V600E prevalence in anaplastic thyroid cancer may partly be explained by differences in the distribution of precursor lesions. Since *BRAF* mutations are more frequently associated with papillary thyroid carcinoma–derived ATC, populations with a higher proportion of PTC-associated ATC may demonstrate higher mutation rates^26,27^. BRAF/MEK inhibitors have been shown overall response rates of about 56–69% across multiple studies with median overall survival reaching up to 14.5 months and estimated one-year survival of up to 80%^10,28–30^. In one of the studies, patients who underwent surgery following *BRAF*-directed therapy had a 1-year OS of 94% with median follow up of 1.21 years^7^. Although, combination regimen with BRAF and MEK inhibitor is preferred, single agent BRAF inhibitor, vemurafenib lead to one patient having a complete response and one partial response in seven ATC patients enrolled in a phase II basket study of *BRAF* V600 mutation-positive nonmelanoma cancers^31^. In a European study done in Portugal, nine out of twenty-seven patients with ATC had *BRAF* V600 E mutation and were treated with dabrafenib and trametinib. Their median overall survival was 15.6 months (475 days), median progression free survival was 8.9 months (270 days) and 71% patients in this group were alive at 12 months. This response was significantly better than for ATCs who were *BRAF* wild type and were treated with multimodal therapy (median PFS less than 32 days and median OS 156 days)^32^.

**Table 1:** Examples of studies using BRAF/MEK inhibitors in anaplastic thyroid cancer

Drug	Number of Patients	Biomarker selection criteria for trial enrollment	ORR (percentage)	Median PFS (months)	Median OS (months)	Comments	References
Dabrafenib (BRAF V600 inhibitor) + Trametinib (MEK inhibitor)	36	Yes; *BRAF* V600E-mutant	56%	6.7	14.5	Phase II study. FDA approved BRAF/MEK inhibitors after this study.	^10^
	16	Yes; *BRAF* V600E-mutant	69%	Not reached	Not reached	Retrospective study. DOR, PFS, OS not reached due to lack of events; 12-month estimates of 90%, 79%, and 80%, respectively.	^29^
	17	Yes; *BRAF* V600E-mutant	82%	4.7	6.9	Retrospective study.	^28^
	6	Yes; *BRAF* V600E-mutant	50%	5.2	9.3	Retrospective study. All patients positive for *BRAF* V600-E	^6^
	11	Yes; *BRAF* V600E-mutant	82%	7.4	10.4	Phase II study.	^30^
	9	Yes; *BRAF* V600E-mutant	N/A	8.9	15.6	71% patients alive at 12-mo.All patients had CR or PR, though 4/9 had progression following PR.	^32^
Vemurafenib (BRAF V600 inhibitor)	7	Yes; *BRAF* V600-mutant	29%	Not determined	Not determined	Retrospective study. 1 CR 12.7 months,1 PR death at 15.5 months	^31^

*DOR* duration of response, *ORR*overall response rate, *OS* overall survival, *PFS* progression-free survival.

### Immunotherapy (Table 2)

Immunotherapy for treating ATC involves the use of immune checkpoint inhibitors (ICIs). These are antibodies that block programmed death receptor-1 (PD-1) and cytotoxic T-lymphocyte associated protein-4 (CTLA4) on T-cells and programmed death-ligand 1 (PD-L1) on tumor cells, thus enhancing immune system’s ability to identify and control or shrink tumor cells. Certain tumors have a better response to ICIs than others and are considered more immunogenic. One of the biomarkers of response to ICI is expression of PD-L1 on tumor cells, though the predictiveness of this biomarker has been debated^33–35^. This is primarily determined by immunohistochemistry (IHC) based PD-L1 expression. However, for ATC, a defined score or optimal cut off for choosing PD-L1 or PD-1 positive samples are not known. Small studies have investigated PD-L1 expression in ATC patients and results are variable. In a retrospective study 43% of 30 patients had PD-L1 expression by IHC (median percentage staining for PD-L1 in tumor cells was 33%). PD-1 staining on surrounding inflammatory cells was noted to be 40% of ATC cells^36^. In another study with 49 ATC specimen, 28.6% was positive for PD-L1 (PD-L1 expression in ≥5% of the tumor cell samples by IHC)^37^. While in a different analysis 70–90% patients were positive for PD-L1 by IHC (cut off for positivity of PD-L1 was if ≥25% of the cells were positively stained)^38^. *PD-L1* amplification was studied in a large tumor database, and 9 out of 177 ATC samples (5.1%) had *PD-L1* amplification^39^.

**Table 2:** Examples of studies with use of immunotherapy-based strategies in anaplastic thyroid cancer

Drug	Number of Patients	Biomarker selection criteria for trial enrollment	ORR (percentage)	Median PFS (months)	Median OS (months)	Comments	References
Spartalizumab (PD-1 inhibitor)	42	No	19%	1.7	5.9	Phase II study.	^47^
Pembrolizumab (PD-1 inhibitor) or Nivolumab (PD-1 inhibitors)	13	No	16%	1.9	4.4	Retrospective study. 10 patients positive for PD-L1, and 7 of these also had a *BRAF* V600E mutation.	^48^
Nivolumab (PD-1 inhibitor) + Ipilimumab (CTLA4 inhibitor)	10	No	30%	N/A	N/A	Phase II study.	^50^
Nivolumab (PD-1 inhibitor) + Ipilimumab (CTLA4 inhibitor)	4	No	25%	N/A	24+	Phase II study (DART)	^51^
Durvalumab (PD-L1 inhibitor) + Tremelimumab (CTLA-4 inhibitor)	12	No	0%	N/A	3.3	Pilot study. 1 patient alive beyond 1 year	^49^
Nivolumab (PD-1 inhibitor) + Ipilimumab (CTLA4 inhibitor)	10	No	30%	4.3	N/A	Phase 2 nonrandomized clinical trial. With a median follow-up of 22.2 months (0.5–26.1 months), the median OS was not reached.	^52^
Atezolizumab (PD-L1 inhibitor) + vemurafenib (BRAF inhibitor) + cobimetinib (MEK inhibitor)	19	Yes, *BRAF* variant	50%	13.9	43	Prospective, non-randomized, phase II trial.	^12^
Atezolizumab (PD-L1 inhibitor) + cobimetinib (MEK inhibitor)	21	Yes, RAS, NF1 OR NF2 variant	14%	4.8	8.7		
Atezolizumab (PD-L1 inhibitor) + bevacizumab (VEGF inhibitor)	3	Yes (no *BRAF, RAS, NF1* or *NF2* variant allowed)	33%	1.3	6.21		
Pembrolizumab (PD-1 inhibitor) + lenvatinib (Multikinase inhibitor)	6	No	83%	16.5	18.5	Retrospective study. All patients had positive PD-L1 expression	^55^
Pembrolizumab (PD-1 inhibitor) + lenvatinib (Multikinase inhibitor)	27	Yes (no *BRAF* mutation allowed)	51.9%	9.5	10.25	Phase II study. Without *BRAF* mutation	^56^
Pembrolizumab (PD-1 inhibitor) + lenvatinib (Multikinase inhibitor)	36	Yes, *BRAF*-wild type	36%	7.7	6.2	In evaluated specimen, PDL-1 CPS ≥ 1 in 96% (27/28) patients. Median duration of treatment 16 mo	^57^
Pembrolizumab (PD-1 inhibitor) + lenvatinib (Multikinase inhibitor) or trametinib (MEK inhibitor) or dabrafenib (BRAF inhibitor) + trametinib (MEK inhibitor)	12	No	42%	2.96	6.93	Retrospective study. Pembrolizumab added as salvage therapy to kinase inhibitors. OS and PFS is calculated from the start of pembrolizumab	^58^
Anlotinib (multi-target TKI) + Sintilimab (anti-PD-1)	18	No	44.4%	7.5	12.9	Prospective study	^59^
Atezolizumab (PD-L1 inhibitor) + Nab-paclitaxel or Paclitaxel (Taxane chemotherapy)	9	Yes (no *BRAF, RAS* or *NF* mutation allowed)	11%	2.73	4.4	Phase II trial, patients had a contra-indication to anti-VEGF therapy (such as high risk for bleeding or fistula)	^60^

*N/A* not available, *ORR* overall response rate, *OS* overall survival, *PFS* progression-free survival.

Since PD-L1 expression varies widely in ATC and scoring methods are not standardized, PD-L1 alone is an unreliable predictor of response to immune checkpoint inhibitors. Other aspects of tumor biology, including mutational burden, may also influence outcomes. ICI have shown efficacy in highly mutated cancers such as melanoma, and currently approved for any tumor with high tumor mutation burden (high-TMB) defined as TMB ≥ 10 mutations/Mb of sequenced DNA (based on KEYNOTE-158 study)^40–42^. Although many ATCs do not meet the threshold of high TMB, as compared to well differentiated thyroid cancers, ATC has greater mutational complexity and thus may derive benefit from immunotherapy^43–46^.

An early trial to evaluate PD-1 blockade in ATC involved 42 patients who were treated with spartalizumab (anti-PD-1) had an ORR of 19%. About 70% patients were positive for PD-L1 by IHC ( ≥ 1% positive in tumor cells) and median TMB was low at 3.78 mutations/Mb. The response rates were highest (ORR 35%) in patients with PD-L1 ≥ 50% and no RECIST responses was observed in patients with PD-L1 expression < 1%. Also, survival rates were higher if patients were PD-L1 positive; 1-year PFS was 0%, 20%, 29% for PD-L1 expression <1%, 1–49%, and ≥ 50%, respectively. Likewise, median OS was 1.6 months in patients with PD-L1 < 1% compared with not reached for PD-L1 positive patients^47^. A phase II trial that enrolled 13 patients who either received pembrolizumab or nivolumab (both PD-1 inhibitors) showed an ORR of 16%^48^. In another trial pembrolizumab showed an objective response in 3/5 patients (ORR 60%). (NCT02688608)

Combination immunotherapy has also been studied. One of the studies used durvalumab (PDL-1 inhibitor) in combination with tremelimumab (CTLA-4 inhibitor) in 12 patients with ATC along with stereotactic body radiotherapy (SBRT). Median OS was 14.5 weeks and one patient was alive after one year^49^. Two other studies showed rather positive results. Nivolumab and ipilimumab (CTLA-4 inhibitor) had a response rate of 30% in ten ATC patients and two of the patients were disease-free at 26 and 13 months after start of treatment^50^. Another study investigating the nivolumab and ipilimumab combination included four ATC patients and one of these patients had a response lasting more than two years^51^. A recent phase 2 trial utilized dual checkpoint inhibition with nivolumab and ipilimumab in a cohort of 10 patients with ATC. These patients’ tumors had either progressed or the patients had unacceptable toxicities from prior therapies or were not candidates for kinase inhibitors. In this study, ORR was 30%. Median PFS was 4.3 months and median OS was not reached but both 12-month OS and 24-month OS rates were 66.6%^52^.

PD-1/PD-L1 axis inhibitors may potentiate the effect of BRAF inhibitors and together significantly decreased tumor volume as compared to either therapy alone based on ATC mouse models^53^. A recent trial published remarkable results with the combination of atezolizumab (PD-L1 inhibitor) with targeted therapy. The cohort of 19 patients with *BRAF V600E* mutation who received combination of atezolizumab with vemurafenib/cobimetinib (BRAF/MEK inhibitors) had a median OS of 43 months and PFS of 13.9 months. Responses in some patients were so good that they underwent complete tumor resection. It was noted in the study that 27/30 patients were PD-L1 positive with tumor proportion score (TPS) of 5% or higher but there was no correlation between PD-L1 expression and OS^12^.

In murine models, PD-L1 therapy has shown to enhance lenvatinib’s efficacy by modifying tumor immune microenvironment^54^. A retrospective study with six patients treated with pembrolizumab and lenvatinib demonstrated complete remission in four patients (overall response rate of 66%). Three patients were alive without relapse at 19, 27, and 40 months^55^. Preliminary results of a German phase II clinical trial (ATLEP) using the same regimen were presented at European Society for Medical Oncology demonstrating a response rate of about 52%. The median progression free survival was approximately nine months and overall survival about ten months. Notably, 7 out of 27 patients lived longer than two years^56^. A study in the USA using this combination in 36 patients with metastatic *BRAF* wild type ATC resulted in a PFS of 7.7 months and OS of 6.2 months while ORR was 36% and median duration of response was 16 months^57^.

Another study analyzed the impact of adding checkpoint inhibitors as a salvage therapy in twelve patients whose disease progressed while on kinase inhibitors. Median OS from start of TKI was 10.43 months while median OS and PFS after addition of pembrolizumab was 6.93 and 2.96 months. Five of 12 patients attained a partial response. It was concluded that pembrolizumab might be an effective salvage therapy when progressing on TKI^58^. A rather recent prospective study demonstrated favorable outcomes with combination of anlotinib (a novel, multi-targeted TKI) and sintilimab (PD-1 inhibitor) in 18 patients with locally advanced or metastatic ATC. ORR was 44.4%, median PFS was 7.5 months, median OS was 12.9 months while 1-year OS rate was 61.1% and 8/18 patients were still alive without progression at the end of follow up (median follow up time was 14.6 months)^59^. Another phase II trial explored adding taxane-chemotherapy to immunotherapy w atezolizumab in patients who had contra-indication to antiangiogenic (anti-VEGF) drugs and who did not harbor *BRAF, RAS* or *NF* mutations found out that this combination was safe but offered limited survival benefit as compared to immunotherapy alone^60^.

Immunotherapy with pembrolizumab in combination with dabrafenib and trametinib was recently used in neo-adjuvant setting for *BRAF* V600E-mutated stage IVB/IVC ATC in a phase 2 trial with 42 patients. Following neoadjuvant therapy, 30/42 (83%) patients were able to undergo surgery. Out of these, 20/30 (67%) had complete pathological response while the rest had residual ATC in the resected specimen. Select patients also received adjuvant radiation therapy to neck and adjuvant pembrolizumab, dabrafenib, and trametinib. Median OS was 20 months and median PFS was 13.9 months^61^. The results of this study are encouraging. Multiple other clinical trials studying immunotherapy in ATC are currently in progress.

### Other targeted therapy (non BRAF/MEK inhibitors) (Table 3)

Studies have shown ATC has multiple biological targets that can be inhibited by tyrosine kinase inhibitors^62^. Thus, targeted therapies have been widely used to treat ATC. Lenvatinib is a multi-kinase tyrosine inhibitor that has been approved in Japan since 2015 for treatment of ATC patients. Studies have shown its efficacy including a phase 2 trial that showed an OS of about 10.6 months with use of lenvatinib in ATC patients^63^. However, disappointing results were noted in two studies; one reported estimated 1-year overall survival rate of 11.9% while the other was terminated early as the predefined response threshold (ORR of 15%) was not reached on interim analysis^64,65^. Overall, most studies show an OS of 3–5 months with the exception of one retrospective study where median OS was 10.2 months^66–73^. The reason for heterogenicity of outcomes is not clear but it may be due to patient selection and disease characteristics in these studies. One French study found that patients with mixed ATC had significantly longer response to lenvatinib as compared to pure ATC (OS 6.3 vs 2.7 months, *p* = 0.026)^69^.

**Table 3:** Examples of studies with the use of non-BRAF/MEK targeted therapy in anaplastic thyroid cancer

Drug	Number of Patients	Biomarker selection criteria for trial enrollment	ORR (percentage)	Median PFS (months)	Median OS (months)	Comments	References
Lenvatinib (Multikinase inhibitor that targets VEGFR, FGFR, PDGFR, RET & KIT)	17	No	24%	7.4	10.6	Phase II, open label study	^63^
Lenvatinib	10	No	30%	N/A	4.75	Retrospective study.	^66^
Lenvatinib	14	No	28.6%	5.7	6.7	Retrospective study.	^67^
Lenvatinib	11	No	27.3%	N/A	N/A	Retrospective study.	^68^
Lenvatinib	10	No, see comments	30%	2.7	3.9	Retrospective study. Eight *BRAF* wild type and two *BRAF* V600E mutant tumors	^6^
Lenvatinib	23	No	9%	2.7	3.1	Retrospective study.	^69^
Lenvatinib	23	No	17.4%	2.5	5.5	Retrospective study.	^70^
Lenvatinib	42	No	11.9%	N/A	N/A	Phase II study.	^64^
Lenvatinib	34	No	2.9%	2.6	3.2	Phase II study.	^65^
Lenvatinib	13	No	23%	3.8	10.2	Retrospective study.	^71^
Lenvatinib	20	No	10%	N/A	N/A	Retrospective study.	^72^
Lenvatinib	32	No	15.6%	N/A	3.2	Retrospective study.	^73^
Sorafenib (Multikinase inhibitor targeting Raf-Ras pathway, VEGF pathway, and FLT3)	20	No	10%	1.9	3.9	Phase II study.	^74^
Imatinib (Tyrosine kinase inhibitor targeting BCR-ABL, c-KIT, and PDGFRA)	8	Yes, PDGF receptor expression positive by immunohistochemistry	25%	N/A	N/A	Phase II study.	^75^
Sunitinib (Multikinase inhibitor that inhibits VEGFRs, PDGFR, CSFR and c-KIT)	4	No	N/A	9.8mo	N/A	Phase II study.	^76^
Pazopanib Multikinase inhibitor, angiogenesis inhibitor that targets VEGFR-1, -2 and -3, PDGFR-α and -β, and c-kit.	15	No	0%	2.1	3.7	Phase II study.	^77^
Apatinib (Tyrosine kinase inhibitor that binds and inhibits VEGFR2)	8	No	37.5%	N/A	N/A	Phase II study, 3 patients had SD and 3 had PR.	^80^
Gefitinib (EGFR inhibitor)	5	No	0%	N/A	N/A	Phase II study. One patient had 32% reduction in tumor volume that did not meet criteria for partial response	^78^
Larotrectinib (Tropomyosin receptor kinase inhibitor)	7	Yes; TRK fusion-positive	29%	2.2	8.8	Phase I/II study.	^79^
Everolimus (mTOR inhibitor)	7	No	14.3%	2.2	4.6	Phase II study. PI3K/mTOR/Akt-mutated ATC subgroups appeared to benefit from everolimus	^83^
Everolimus (mTOR inhibitor)	6	No	N/A	~2.5	~3.3	Phase II study.	^81^
Everolimus (mTOR inhibitor)	5	No	N/A	N/A	7.4	Case series. One patient had a partial response for 27.9 months	^84^
Fosbretabulin (Combretastatin A4 phosphate (microtubule destabilizing agent))	26	No	14.8%	N/A	4.7	Phase II study.	^85^
Efatutazone (peroxisome proliferator-activated receptor gamma (PPARγ) agonist) and Paclitaxel	15	No	14.2%	2.2	4.6	Phase I study.	^86^

*N/A* not available, *ORR* overall response rate, *OS* overall survival, *PFS* progression-free survival, *PR* partial response, *SD* stable disease.

Other kinase inhibitors studied in ATC did not show favorable responses. These include sorafenib, imatinib, sunitinib, pazopanib, gefitinib, and larotrectinib^74–79^. Apatinib, a VEGFR-2 inhibitor was studied in eight patients with ATC; three patients achieved partial response while one continues treatment for 73 weeks. However, two patients in the study had to stop treatment at 15 weeks and 13 weeks due to intolerable toxicities^80^.

Since, PI3K/mTOR/AKT pathway has also been noted to be involved in pathogenesis of ATC, Everolimus, an mTOR inhibitor has been studied in ATC. However, no significant changes in outcome were noted for most patients with a few exceptions with excellent responses^81,82^. In a study of seven patients, one patient had PFS of 17.9 months and another had stable disease for 26 months^83^. In another study, a patient had a partial response for 27.9 months^84^.

Other targeted therapy agents that have been studied in ATC but did not show statistically significant improved responses include fosbretabulin (combretastatin CA4P, a vascular disrupting agent) and efatutazone (an oral PPAR-γ agonist)^85,86^.

### Role of precision oncology in management of ATC

Keeping in view above literature, it is recommended all patients with ATC should get molecular testing as soon as they are diagnosed. This can be done by PCR assay or IHC for *BRAF* V600E to yield quick results. Furthermore, next-generation sequencing (NGS) must be sent for comprehensive molecular profiling as well as to understand tumor mutational burden and microsatellite instability status. IHC for PD-L1 and HER-2 should also be undertaken. Profiling can be done on core biopsy or FNA especially for patients who are not undergoing surgery. If samples are not available cell-free DNA assay from peripheral blood could also be considered. If molecular testing is not available, patients should be referred to centers that have these facilities as so they are able to get biomarker directed therapies or enroll in clinical trials^87^.

## Illustrative case

A 60-year-old man presented with left neck mass. Tissue biopsy revealed back-to-back follicular structures focally with cytologic features suggestive of papillary thyroid carcinoma. IHC stains showed positive staining of TTF-1 and thyroglobin in the majority of the tumor and negative for Napsin-A. The differential diagnosis included a follicular neoplasm or a follicular variant of papillary thyroid carcinoma. A < 1.0 mm focus of atypical cells with a more solid growth pattern was also present that was negative for TTF-1 and positive for cytokeratin. This raised concerns of poorly differentiated carcinoma in the background of thyroid neoplasm, although no mitotic figures or necrosis were identified. He underwent modified radical neck dissection. Pathology was reported to be papillary thyroid carcinoma with a small component, about 10% demonstrating high-grade features raising concerns of anaplastic transformation. Two months later he underwent total thyroidectomy, that showed papillary thyroid cancer. Within a month, he had recurrent disease and underwent left neck mass excision. This time pathology revealed anaplastic carcinoma.

PET/CT a month later showed progression with pulmonary metastasis and lymph node involvement. Patient was started on lenvatinib and had mixed response with dramatic decrease in neck mass but new adrenal metastasis on a follow up CAT scan. He developed respiratory failure at this time due to rapidly evolving metastatic disease. He was hospitalized, intubated, and required mechanical ventilation. Lenvatinib was stopped and patient was enrolled on MyPathway clinical trial *BRAF* V600 mutation arm utilizing vemurafenib and cobimetinib^88^. A month later, cobimetinib had to be held due to decreased cardiac reserve. Repeat imaging in a few days showed worsening lung and adrenal metastasis so he was taken off the trial. Since, on exam, the neck mass was regressing, vemurafenib was continued and lenvatinib was added based on previous favorable response. Patient’s clinical status started to improve but one month after starting lenvatinib, he was found to have brain metastases. Since he was still in the intensive care unit, radiation-oncology team did not recommend radiation therapy.

At that point, immune checkpoint inhibition was considered as immunohistochemical analysis of the tumor tissue demonstrated strong PD-L1 expression (TPS 100%). This biomarker finding supported the potential role of immune checkpoint blockade as part of a precision oncology based therapeutic strategy. The patient was started on nivolumab, a PD-1 inhibitor. This report followed the guidelines of the IRB-approved UCSD- Profile Related Evidence Determining Individualized Cancer Therapy (PREDICT) study (NCT02478931) and any investigational studies/agents for which the patient gave consent^89^.

**Fig. 1:**
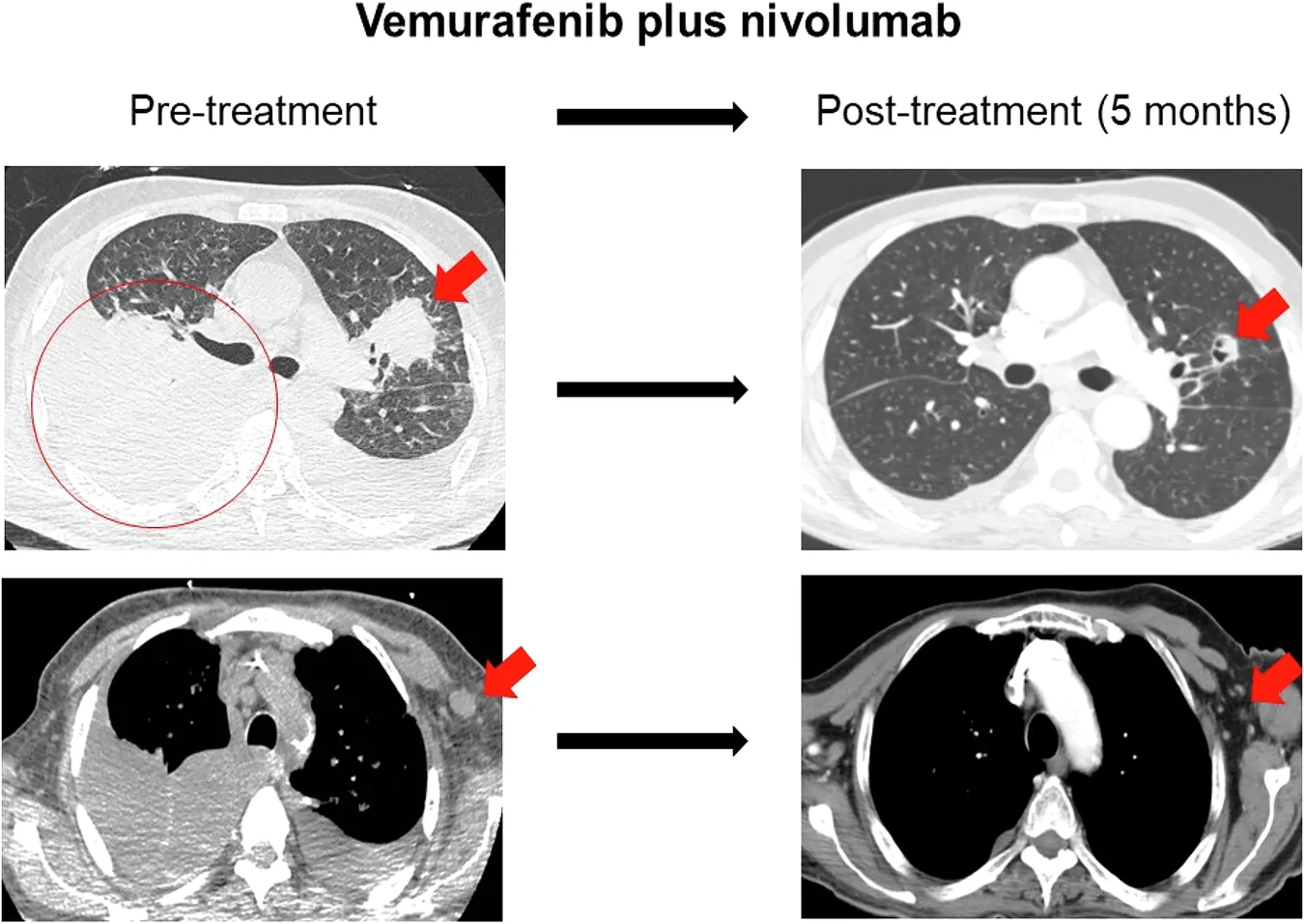
A 60-year-old male patient intubated and admitted to ICU with complications due to ATC was started on vemurafenib (BRAF inhibitor) (based on *BRAF* V600E mutation) and added nivolumab (PD-1 antibody) (based on PD-L1 immunohistochemistry of 100%) about a month after initiation of vemurafenib. CT at 5 months showed response including intrathoracic LN, pulmonary nodules (red arrow) as well as improvement in pleural effusion (red circle). The patient achieved a partial remission. Patient was extubated and discharged home with PFS of 9 months.

Shortly thereafter, lenvatinib had to be stopped due to a perinephric hematoma. This was re-introduced after a month when aspirin and heparin was stopped. Imaging done one and a half month after starting nivolumab, about five months after vemurafenib and with on and off lenvatinib showed marked improvement of his disease overall. Since he did well clinically (extubated, ambulating), he was discharged on nivolumab and vemurafenib. About four months later, he was performing his daily activities by himself and imaging showed continued regression and imaging showed continued regression (Fig. 1). Progression-free survival was 9 months.

This case shows that a personalized biomarker-based treatment plan that is tailored to individual patients’ disease may improve patient outcomes. A similar response was noted in another case report with vemurafenib and nivolumab where the patient with ATC continued to be in remission 20 months after starting nivolumab^90^. A recent study utilized the approach of dual biomarker-matched targeted and immunotherapy (each matched to specific molecular aberrations) in 17 patients with advanced refractory cancers: disease control rate (includes stable disease of at least six months/complete and partial remission) was 53%, median PFS was 6.1 months and median OS was 9.7 months. Three patients had prolonged ongoing survival ranging from 23+ to 62+ months^91^. Furthermore, in vitro drug evaluation via cell viability studies has also been used to assess response of a targeted treatment. This strategy was demonstrated in a patient with ATC, status post total thyroidectomy, whose tumor tissue was being cultured while he received radiation/chemotherapy. Cell viability assays using different drugs identified sorafenib to have the most pronounced effects and treatment with the drug resulted in eradication of disease ongoing at 43 months since initial diagnosis^92^.

## Conclusion

ATC is an extremely aggressive disease, unlike many other types of thyroid cancer. The median survival from diagnosis ranges from up to 10 months for patients who are able to get surgery to three months for inoperable cases^1^. About 50% patients have widely metastatic disease at the time of diagnosis, while 40% have extrathyroidal invasion with or without lymph node metastasis^4^. Treatment with cytotoxic agents has not been shown to significantly improve outcomes. In a recent meta-analysis, it was discovered that 33% of these tumors carried *BRAF* V600E mutations^93^. The ORR in patients who have *BRAF* V600 mutation in ATC to BRAF and/or MEK inhibitors is 56–69%^10,29^. In addition, about 70% of patients express PD-L1^47^. Response to immune checkpoints inhibitors (anti-PD-1, anti-PD-L1, CTLA-4) is variable with ORR ranging from 19 to 75%^45^. Combination of targeted therapy and immune-therapy has also shown benefit. With these novel approaches, an improvement in overall survival has been noted in recent years for patients with ATC^7^. We present an illustrative patient with terminal widely metastatic anaplastic thyroid cancer (who was on a ventilator) who had a robust partial remission and recovery lasting nine months on biomarker-based therapy with nivolumab and vemurafenib. Further studies using biomarker-based gene- and/or immune-targeted agents are warranted in aggressive anaplastic thyroid cancer.

## Data Availability

No datasets were generated or analysed during the current study.
